# Analysing Staff Perspectives on Parent/Carer Partnerships in an English Trauma Informed School

**DOI:** 10.1007/s40653-026-00849-8

**Published:** 2026-04-01

**Authors:** Aisling Culshaw

**Affiliations:** School of Education, Liverpool John Moores University, Liverpool, UK

**Keywords:** Trauma informed practice, Parent partnerships, Relational pedagogy, Marginalisation in education, Staff support

## Abstract

This study analyses staff perspectives on parent/carer partnerships in a trauma informed primary school in Northwest England. The research draws from broader research on trauma informed practices in education but focuses specifically on one emergent theme: Parent/Carer Partnerships. Drawing on semi-structured interviews with staff at a school characterised by high levels of deprivation, complex needs and a strong and embedded trauma informed ethos, the study analyses how partnerships with parents/carers are created and sustained. The study’s findings are discussed in relation to existing literature on trauma informed practice in education and concludes that staff adopt holistic, culturally aware and emotionally attuned approaches that position parents/carers as paramount in supporting children’s learning and development. However, these efforts are often constrained by systemic pressures such as educational bureaucracy and performativity pressures. The study argues for deeper, contextually informed engagement with families as key to effective trauma informed practice in education, where sustained investment in staff training and support enables and advocates for traditional, deficit-based assumptions towards vulnerable children and their families to be challenged. The study contributes to a growing evidence base on the central role of schools in supporting vulnerable families through inclusive, restorative and relational practices, key aspects of practice that is trauma informed.

**Supplementary Information:**

The online version contains supplementary material available at https://doi.org/10.1007/s40653-026-00849-8.

## Introduction

Developing and maintaining strong, trusting relationships between families and schools is essential in supporting the wellbeing of children, particularly those who have had or continue to have experiences of trauma and adversity. Whilst international and policy frameworks promote ‘parent/carer partnerships’ (OECD, 2012; Department for Education, 2024a) there exists limited research that analyses how staff navigate these partnerships in schools, particularly in relation to vulnerable children. For children who experience complex home lives, other adults, such as staff in schools, can become the most consistent figures in their lives (Treisman 2016, 2017).

This study draws from wider research that focuses on how current behaviour management approaches in schools often rely on punitive systems that risk further marginalisation for vulnerable children and their families. Research indicates the commonality of adversity and trauma in schools, where almost 50% of children will have experiences of at least one form (Young Minds, 2021). For some children, their vulnerabilities might be evident in, for example, their characteristics, demographics, or parenting (Fondren et al. 2020) and can manifest on how they behave in schools. As a result of an increased struggle to adhere to the routines and boundaries of school, vulnerable children may be more likely to become subject to behaviour management procedures that position their behaviour as problematic, rather than a communication of an unmet need (Hebert, 2018; Timpson, 2019; Weissman, 2015). For these children, adverse experiences become the impetus for feeling othered or outside of accepted societal practices, where their inability to abide by the school rules and boundaries is perceived as their fault, setting the tone for feeling othered within society.

As Forner (2017) states, in times of need, children, “cannot get themselves back from the back roads” (70) without adult support. This conceptualisation illuminates the central role of adults, both parents and educators, in helping children to feel safe and valued in school where staff are uniquely placed to provide not only academic guidance, but also relational support to children and their families. Sherwood (2017) asserts how school staff can become a reservoir of hope for vulnerable children, but this is often undermined by systemic pressures of bureaucratic expectations and performative demands. In England, this is evident in national guidance, such as the Behaviour in Schools document (Department for Education, 2024a) which appears to prioritise compliance and behaviour management over mutual respect, connection and person-centred practice. Against this backdrop, the study is guided by the following research question – *What are school staff perspectives on the value of parent/carer partnerships within a trauma informed school?* The paper will focus on how school staff experience and interpret partnerships with parents/carers. The research question is guided by aims to explore what shapes staff approaches in working with parents/carers and how these partnerships influence broader school culture and family outcomes. To investigate these themes, a qualitative case study was undertaken in a primary school that identifies as trauma informed. Semi-structured interviews were conducted with a purposive sample of school staff with teaching and pastoral support roles. An interpretive approach was employed, and data was analysed using Braun and Clarke’s reflexive thematic analysis (2006, 2019) where the authors own positionality as a researcher and educator informed the process.

The findings suggest that prioritising mutual respect, empathy and collaboration with parents/carers is vital in creating an inclusive and trauma informed school environment, concepts that are foundational to positive behaviour support in both home and schools. As such, acknowledging and nurturing the role of parents in schools is essential. The study contributes to a growing body of research that challenges punitive behaviour systems and calls for more compassionate, person-centred approaches in schools (McCluskey, 2018; Mullet, 2014; Zehr, 2002). It complements further the shift towards home school partnerships through better understanding of how staff perceive connections with parents/carers.

## Literature Review

Practice that is trauma informed recognises that adversity in childhood, such as abuse, neglect or insecure attachments can have significant and lasting effects on children’s learning, development and behaviour (Office for Health Disparities 2022). Children who have experienced adversity or trauma in their lives often struggle to form trusting relationships with adults which can result in displays of behaviour that can be perceived as challenging or disruptive, characteristics of the fight, flight, freeze response (McCarty, 2016). In schools, the response that school staff take to such behaviour becomes critical, where their perspectives are the catalyst to what happens next. Sherwood (2017) defines trauma informed practice as a proactive rather than reactive approach, where staff must understand the drivers of behaviour in order to respond appropriately. As such, staff relationships have a profound effect on children’s capacity to feel a sense of belonging in schools (Blitz et al., 2020).

Trauma informed practice is further strengthened when school staff work to build positive, empathetic relationships with not just the children they work with but also their families. Clarke et al. (2010) posit that effective partnerships are founded upon mutual respect, which means moving beyond the transactional parent teacher communication and towards authentic, trusting partnerships. Martin and Collie (2019) highlight that when school staff demonstrate consistency and care in their relationships with parents/carers, they create an environment where children and their families feel a sense of belonging and acceptance.

Staff perceptions of trauma and its effects will influence how behaviour is understood and responded to in schools. Sherwood (2017) and Jensen (2009) argue that children’s behaviour must be perceived as a form of communication, particularly for vulnerable children. Burke-Harris (2018) and Jurkovic (1998) describe how children who have experienced trauma and adversity are more likely to display behaviours such as hypervigilance or a lack of ability to abide by school routines and boundaries. Blitz et al. (2020) found that when staff had been provided with the training and guidance to adopt relational responses to behaviour, their responses centred upon restoration, reflection and explanation, and in turn promoted a more positive and empathetic school climate. Such insights promote a shift away from punitive, zero-tolerance approaches which are deemed counterproductive for vulnerable children (Bell et al., 2021; Huang & Cornell, 2021).

Parent/carer involvement in children’s education has long been associated with improved outcomes. Ceka and Murati (2016) and Hornby and Lafaele (2011) assert that effective home school partnerships are vital to children’s wellbeing and academic outcomes. This is further supported by Froiland and Davison (2014) and Jeon et al. (2021) where it is evidenced that parent/carer engagement enhances children’s motivation and also helps parents to have a more favourable view of schools. Despite this, many schools continue to face ongoing difficulties in fostering meaningful connections with families. Research (Hornby, 2010; Hornby & Blackwell, 2018) denotes performativity pressures resulting in time constraints and a lack of staff knowledge and understanding as key attributes to this ongoing issue.

Seeking to gain the perspectives of staff, the central aim of this study, is particularly useful in better understanding relational approaches that support or inhibit effective parent/carer partnerships, particularly when typical educational practice and policy is reflective of middle-class expectations (Adele, 2017), making it harder for schools to connect with families from more diverse or disadvantaged backgrounds. This mirrors findings by Pote et al. (2019) who found that informal, community-based parenting support is often more effective and sustainable than structured programmes. Informal support in this context can include early intervention opportunities for parents/carers to connect with the school community, such as drop-in sessions, baby and toddler groups or coffee mornings led by school pastoral or family support staff. Furthermore, the OECD (2012) notes that parent/carer engagement can increase their confidence and reduce hierarchical power imbalances in more formal parenting programmes.

The importance of consistency in trauma informed practice is necessary to support all those involved in the school community, staff, children and families alike, where inconsistencies can ignite anxiety and confusion (McGrath & Van Bergen, 2015). Research repeatedly evidences that children thrive in spaces where key adults are consistent, behave predictably and with compassion (Lopes et al., 2017; Martin & Collie, 2019). As such, trauma informed practice must be embedded at a whole-school level and not left to goodwill or staff preference (Thomas, 2021).

Trauma informed practice should not be perceived as a ‘one-size-fits-all’ where aspects such as cultural consideration must be applied (Office for Health Disparities 2022;Irby & Clough, 2015; Anfara et al., 2013; McCluskey, 2018), for example flexibility and cultural sensitivity, such as complex challenges faced by families due to experiences of trauma and adversity (Blitz et al., 2020). Addressing deficit views towards deprivation is also a key factor of trauma informed practice where Gershenson et al. (2016) posits that teacher expectations are often shaped by unconscious bias, leading to lower expectations or pathologising of working-class families. McAra and McVie (2010) states that these views are often reinforced through institutional practices in society, such as schools, where Weissman (2015) asserts that it is imperative that school staff engage in reflective practice to avoid reinforcing such social hierarchies. Bates (2021) calls for ongoing professional development to support school staff to become more knowledgeable on trauma and its effects and in turn develop a wider capacity to understand behaviour as a form of communication. Jones (2022) cautions that in current educational practice, initiatives that centre on nurture and restoration depend heavily on the goodwill of school staff, rather than it being embedded in school policy and practice at government and institutional level. Skovdal and Campbell (2015) state that trauma informed practices require collaboration between schools, families and wider services and that school staff are key agents in facilitating and maintaining these partnerships.

## Methodology

The study aimed to analyse how school staff perceive parent/carer partnerships within the context of a trauma informed school. In 2022, the school involved was praised by Ofsted, the English school inspectorate, for its inclusive and relational ethos. The study sought to investigate how a trauma informed approach informs staff engagement with families, particularly in terms of shaping school culture and in promoting positive behaviour. Given the study’s aim, an indictaive qualitative research design was employed.

Trauma informed practice is founded upon a relationship driven framework (Office for Health Disparities 2022; McCluskey, 2018) which in turn places value on how those involved perceive and construct its meaning and implication. Therefore, a constructivist epistemology underpinned the study (Guba & Lincoln, 1989). This enabled analysis of how school staff perceive their relationships with parents/carer and its influence on the broader school environment. Situated in the belief that reality is constructed through one’s experiences and interactions (Bryman, 2012), the study did not seek to finalise best practice but instead to highlight how staff from one school navigate and define these partnerships.

## Research Design

A case study design was selected to enable an in-depth exploration of staff perspectives on parent/carer partnerships in a trauma informed school which also allowed for consideration of broader social and contextual influences (Gillespie, 2023). Case study methodology complements small-scale research when the aim is to contribute nuanced insights into a broader body of knowledge rather than to generalise across settings (Cousin, 2005; Bassey, 1999). In this study, the school itself was treated as the case, with the interviews providing one source of insight into its wider relational culture and organisational context. Validity was ensured through applying triangulation through engagement with literature and contextual data where, in the following section, a ‘thick description’ of the research school is provided to support, and a reflexive stance was maintained throughout (Stake, 1995; Bassey, 1999).

### School Context

The school involved in the study is in a demographically deprived area of Northwest England. The area is within the most deprived nationally (Ministry of Housing, Communities and Local Government, 2019), with significant challenges related to income, employment, health, education, housing and crime. Research confirms that children from areas of deprivation often face cumulative risks that impede their learning and development (Child Poverty Action Group, 2022; Sherwood, 2017) where due to this context, the presence of adversity and trauma is endemic rather than exceptional.

In response to the needs of the area, the school has developed a school wide trauma informed ethos. Although not mandated by the local authority, leaders elected to prioritise practice that was trauma informed and as a result commissioned training opportunities to support staff understanding of trauma and its effects on children and their families. The school demographic data reinforces the intensity of need where approximately 84% of children fall into vulnerable groups including Special Educational Needs and/or Disabilities (SEND), safeguarding concerns and children who are in receipt of additional funding due to hailing from low-income families.

Due to the level of vulnerability across the school population, the school have been able to use Pupil Premium funds, a UK government initiative aimed at improving educational outcomes for disadvantaged pupils (Department for Education, 2024b) to provide pastoral support, though this remains modest in size given the high levels of complexity and need. School staff are actively supported to practice and promote trauma informed values (Office for Health Disparities 2022) where the school has undertaken extensive training and professional development on trauma informed themes and relational practice. While the school’s move towards trauma informed practice is ongoing, there is a clear commitment to this ethos at whole-school level where leadership support and investment has been central in shaping this direction.

The school context provided a compelling site for exploring staff perspectives on parent/carer partnerships in a trauma informed school. Its response to need due to the demographics of the school, alongside its whole school trauma informed ethos, formed a fitting backdrop to better understand staff perspectives in such a relationally focused school environment.

## Participants and Sampling

The research sample comprised of eight staff members (Table 1), who were selected using non-probability sampling, combining convenience, purposive and stratified techniques. Purposive criteria required that participants worked directly with children or families in the school and were familiar with the school’s trauma informed ethos. To ensure breadth across the case, sampling was also stratified to include representation from Early Years, Key Stage 1 and Key Stage 2, and to capture a balance between teaching and pastoral staff roles, known in the school as Emotional Literacy Support Officer’s (ELSA). Convenience sampling was then applied based on staff availability and willingness to participate. These criteria aligned with the study’s aim of analysing staff perspectives within a single bounded case rather than comparing demographic characteristics across individuals. The table 1 below lists the pseudonyms assigned to staff participants and details their role within the school.

**Table 1:** Participant roles within the school

Staff Name	Role
Sadie	Emotional Literacy Support Officer [ELSA]
Maeve	Early Years Teaching Assistant
Hayley	Key Stage 2 Teacher
Aisha	Key Stage 2 Teacher
Cara	Emotional Literacy Support Officer [ELSA]
Sam	Key Stage 1 Teacher
Layla	Early Years Teacher
Roz	Key Stage 1 Teaching Assistant

## Data Collection

Data was collected through semi-structured interviews, chosen for their ability to support in-depth, participant led dialogue (Knott et al., 2022). Interviews were guided by prompts developed through the literature review (Jimenez & Orozco, 2021) enabling flexibility whilst ensuring resonance to the research aims. These prompts covered broad areas such as staff experiences of engaging with parents/carers, their views on the school’s trauma informed ethos, practices that supported or hindered relationships with families, and wider organisational or contextual factors shaping home school partnerships. This approach provided structure without restricting the discussion, allowing participants to guide the direction and emphasis of the conversation. Each interview lasted up to one hour, was audio recorded with consent, and then later transcribed verbatim. Notetaking was avoided during the interviews to allow for a more comfortable and open exchange (Opdenakker 2006; Muswazi & Nhamo, 2013).

## Ethical Considerations

Ethical approval was granted by the researchers host university and adhered to BERA (2018;2024) ethical guidelines and the Data Protection Act 2018. Informed consent was obtained from all research participants and confidentiality was maintained throughout the study.

## Interpretive Approach and Researcher Reflexivity

The study was informed by an interpretivist epistemology, which assumes that reality is socially constructed and meaning making is shaped through individual’s experiences, interactions and contexts (Guba & Lincoln, 1989). This stance enabled the research to focus on how staff made sense of parent/carer partnerships within a trauma informed school, whilst recognising that such perspectives sit within broader relational and organisational systems (Bryman, 2012).

My positionality as an educator with experience of trauma informed practices shaped the perspective that I brought to the analysis, particularly in how I understood and interpreted staff responses in the interviews. I remained mindful, throughout the data analysis process, of how my professional background and assumptions might influence the development of codes and themes, noting where my expectations diverged or aligned with participants accounts (Kivunja & Kuyini, 2017). This supported an analysis that stayed rooted in participants’ accounts while being transparent about my role in interpreting the data. The data analysis process applied was Braun and Clarke’s Reflexive Thematic Analysis (2006;2019), selected as it aligns closely with interpretivist principles by acknowledging the researcher as an active contributor to meaning making in research, rather than an impartial observer.

### Reflexive Thematic Analysis

The semi-structured interview data was analysed using reflective thematic analysis (Braun & Clarke, 2006, 2019), an approach recognised for its capacity to provide nuanced insight into experiences, behaviours and attitudes (Kiger & Varpio, 2020). Reflexive thematic analysis aligns well with interpretivist paradigms that emphasise understanding social contexts through the co-construction of meaning making between researcher and participants (Braun & Clarke, 2006). Braun and Clarke (2013) assert the need for rigorous focus when identifying themes that should represent patterned meanings rather than mere data summaries. To guide the analysis, the six-phase framework proposed by Braun and Clarke (2019) was employed.

Six-phase reflexive thematic analysis process.

1. Familiarisation with Data** –** Following the audio recording and transcription of each interview, transcripts were cross checked against the recording twice. During these initial readings, notes were made on recurrent themes, significant statement and initial reflections.
2. Generating Initial Codes **–** Following a second read through, 66 initial codes were identified which were developed reflexively, acknowledging the researcher’s interpretative role and potential biases (Braun & Clarke 2021) alongside existing literature derived in the literature review.
3. Searching for Themes** –** The initial codes were organised into clusters reflecting similarities and differences, leading to the emergence of preliminary thematic groups.
4. Reviewing Themes** –** The preliminary themes were rigorously reviewed to ensure comprehensive coverage of the data. The entire data set was then revisited to confirm that no relevant data has been overlooked or required additional themes.
5. Defining and Naming Themes** –** No further themes emerged during review, however, existing themes were refined and consolidated into four overarching categories – mutual respect and empathy, leadership and support, parent/carer partnerships; and child-centredness.
6. Producing the Report **–** The four themes formed the foundation for the presentation of findings where direct quotations from the staff interviews were integrated to support each theme, and findings were critically contextualised with literature on trauma informed educational practice.

For the purposes of this report, a specific focus on the theme of Parent/Carer Partnerships is conducted. The intent to focus on one theme was to explore, in greater depth, how school staff conceptualise engagement with parent/carers in a trauma informed school. By sharpening the analytical lens on just one theme it allowed for a more detailed and nuanced understanding of staff perspectives.

## Findings

Reflexive thematic analysis generated four overarching themes which were mutual respect and empathy; leadership and support; parent/carer partnerships; and child-centredness. As this article focuses on parent/carer partnerships, this section presents key findings from the school staff interviews that support the ‘Parent/Carer Partnerships’ overarching theme. The findings uncover complex insights into how staff perceive and enact partnerships with parents/carers in a trauma informed school. The subthemes to follow reflect the analytic interpretation of school staff accounts, with links to wider literature considered in the Discussion section.

- Trust and Empathy.

Trust emerged as a central element in how staff understood their work with families, shaping the tone and depth of their interactions with parents and carers -.

*“The relationship with the parents is just as important*,* getting them to trust us because they could have been in the exact same situation (as children)”. (Aisha)*

Aisha’s comment suggests that staff saw trust building as a relational process that required recognising the experiences families brought with them and adjusting their approach accordingly. The example highlights a relational dynamic where staff support children but also seek longer-term solutions through building relationships with their parents/carers.

This awareness of intergenerational trauma is further alluded to by Layla –

*“Quite a lot of the time they (parents) just need to come in for a cup of tea. Coming in really upset and they have a brew and leave feeling better as they had a chance to vent”. (Layla)*

Such informal interactions were framed as important opportunities for connection, enabling parents/carers to approach the school in a way that felt comfortable and non-judgemental. Staff viewed these moments as part of the routine relational work that helped sustain partnerships over time.

Together, these accounts show that trust and empathy formed the basis of how staff engaged with families and understood their ongoing relationships with them.

- Creating and maintaining inclusive spaces.

Staff highlighted the importance of creating spaces where parents/carers felt welcomed and able to participate without fear of judgement -.

*“some of the parents*,* when they come into the headteachers office*,* they are so nervous. But when we have those open conversations and they see we want to help them*,* it changes”. (Hayley)*

This comment reflects an awareness that formal school spaces can feel intimidating for some parents/carers, particularly those with previous negative experiences of schooling. Staff described consciously working to reduce this sense of hierarchy by adopting an open and supportive approach.

This discomfort appears to be, to some extent, mitigated in further comments, such as through the school’s parent and baby group –

*“Every week has a focus*,* so every week it’s like*,* ‘oh you know when you’re going down the road and looking at the road signs? Well*,* that’s literacy’. You know like in a very gentle way helping parents understand*,*”. (Maeve)*

This informal, strengths-based approach repositions parents/carers as capable contributors to their children’s education where they are guided to see their everyday interactions as educational for their children.

These reflections suggest that creating inclusive spaces was understood as key to establishing positive and sustained relationships with families.

- Cultural Considerations.

Staff also discussed the importance of recognising the diverse cultural and practical needs of families -

*“this morning it just happens to be that several of our refugee families needed support foodwise and because they have halal needs and so on and so forth*,* we found somewhere where they could get food from….we found a place*,* and a lot of the families couldn’t get there because they’re trying to better themselves and they’re going to college*,* So*,* we go and get those things and try and help and support them”*,* (Sadie).*

Here, Sadie, a member of the pastoral team (ELSA), framed practical support as an important way of showing respect for family circumstances and reducing barriers to engagement. Meeting cultural needs was seen as an essential part of ensuring that families felt understood and included within the school community.

These examples show that cultural responsiveness was understood as part of everyday relational work rather than a separate or formalised intervention.

- Relational Practice.

Staff highlighted how children’s behaviour can reflect their experiences and norms -

*“For example*,* like for a child*,* ‘well why are you kicking me out of class for swearing when everybody does it. I’m allowed to swear at home’*,* and I say well*,* ‘that’s absolutely fine. You are allowed to swear at home but when we are here*,* we need to consider the younger children. And you know it’s really putting that kind of perspective”. (Sam)*

*“And I think sometimes*,* as well*,* we treat children like adults when they are children*,* you know*,* and if that’s how they were brought up and the experiences that they have at home*,* and once they get worked up and they’re overwhelmed*,* yeah*,* they’re automatic reaction is to swear cause that’s what they are used to”. (Aisha)*

Sam and Aisha’s reflections resonate with trauma informed principles, where behaviour is understood as a form of communication, rather than a wilful defiance. Staff use reasoning and explanation instead of punitive punishment, which evidences a relational pedagogy that seeks to educate and restore, rather than to exert power and control.

A further staff comment highlights how displays of disruptive or challenging behaviour might be a mechanism to protect –

*“So*,* they are behaving in a certain way because they know*,* well*,* their mum will get called and she will have to come and get them and then they can make sure that mums okay and everything’s ok”. (Roz)*

Staff interpreted these behaviours in the context of children’s wider family responsibilities and emotional concerns, highlighting the importance of considering what might be driving a child’s actions.

These accounts show that relational practice was viewed as central to supporting children and understanding their behaviour within broader family contexts.

- Challenging deficit views of deprivation.

Finally, interviewed staff challenged generalisations about poverty and deprivation -.

*“well*,* we know children in more deprived areas probably do struggle more*,* but there might be a boy who lives in a council estate who is super well supported*,* they might not have money*,* but the family is supportive*,* the child is resilient*,* loved*,* heard*,* listened to*,* read to every night”.(Cara)*.

Cara, a member of the pastoral team (ELSA), highlights staff awareness that socioeconomic status does not necessarily reflect the quality of family relationships or parental commitment. Staff emphasised the importance of recognising strengths rather than making assumptions based on socioeconomic context.

These interpretations suggest that challenging deficit views was an important aspect of how staff approached their work with families.

## Discussion

The study’s findings reveal a strong emphasis on the importance of parent/carer partnerships within a trauma informed school. Interviewed staff demonstrate how they perceive their relationships with parent/carers as crucial to supporting the needs of children, especially those who have experienced adversity or trauma in their lives. The paramount role of parents in their children’s education and development has been extensively evidenced (Early Education 2021; Ceka & Murati, 2016) where the current research aligns with these assertions and expands further by highlighting how staff actively support not only children but their wider family. Staff comments suggest a framework of support where trust is central, where vulnerable families can access support, reinforcing the need to go “beyond education” (Skovdal & Campbell, 2015, p. 8).

The school’s responsiveness to family needs indicates a shift from traditional forms of school practice, where home and school were distinct (Kent et al., 2022), towards a more holistic, person-centred approach, aspects which align with trauma informed principles (Office for Health Disparities 2022). The school staff repeatedly described how the school serves as a safe and welcoming space for children and their families, facilitating parent/carer partnerships through simple but meaningful interactions such as informal conversations to access to support. Staff recognise the importance of small acts of engagement such as the offer of a cup of tea and how this can become a mechanism for relieving stress and forging meaningful relationships. Whilst it can be argued that the simplicity of such interactions might suggest a reductionist response, it can also signify the importance of relational practice in creating inclusive school environments, something that Kearns and Hart (2017) state can be undermined due to subject centric pedagogy and classroom management strategies in teacher education.

Parent/carer partnerships remain a perpetual challenge in schools (Adele, 2017; Hornby, 2010) which is hindered by time constraints due to educational bureaucracy and performativity pressures (Kilinç et al. 2016; Lanas & Brunila 2019). However, the school staff in this study did not reflect on such barriers. A possible explanation, as evidenced in the school’s context, is the presence of a strong pastoral team made possible by targeted funding associated with indictors such as higher than average levels of additional need such as SEN, EAL and lower socioeconomic status. A further explanation might be the school leaders’ commitment to training and ongoing support in trauma informed practices increasing staff confidence and a more informed and compassionate school workforce. This is in contrast to current educational practice where there exists limited mandatory training and ongoing support on the impact of trauma and adversity on the developing child and their families, which therefore limits the potential of school staff having the knowledge to understand how this might impact on behaviour in schools (Kearns & Hart, 2017; Dingwall & Sebba, 2018; Kim et al., 2021a).

The view that home school partnerships are historically shaped by parent/carers experiences of schooling resonates with the findings from Schmid and Garrels (2021), where parental engagement was closely linked to socioeconomic factors. For some parents/carers, entering spaces such as the headteachers office may carry worry and apprehension due to past experiences of that space representing judgement and exclusion, particularly felt by children and families who have had negative experiences of education and who feel culturally or socially marginalised (Weissman, 2015). However, staff from the study school advocate for a welcoming and empathetic environment, which challenges traditional views of symbolic power, such as the headteachers office, and instead works to change relational dynamics which work to build a culture of restoration and community (Clarke et al., 2010; Froiland & Davison, 2014; Jeon et al., 2021).

Further examples of cultural considerations are exhibited in staff comments, for example when addressing the dietary needs and logistical challenges of families from minority groups where such examples demonstrate pragmatic commitment to promote inclusive practices. This resonates with the Office for Health Disparities (2022) working definition of trauma informed practice and the emphasis placed on cultural responsiveness, aspects which are deemed necessary for effective trauma informed practice (Thomas, 2021). Similarly, parent/carer support in extracurricular practice, such as the weekly baby group, illuminates the power of accessible and strengths-based interventions with families. Instead of using formal parenting programmes, which are notorious for low uptake amongst vulnerable families (Pote et al., 2019), school staff work to highlight when parents/carers do well, promoting confidence and in turn further engagement (Hornby & Lafaele, 2011; OECD, 2012).

Interviews with staff also highlighted efforts to contextualise behaviour regarding differing home and school norms where staff explain the expectations of school without condemning children’s home lives. These practices align with Sherwood (2017) and Blitz et al. (2020), who argue for relational understanding over strict zero-tolerance rules and boundaries. Furthermore, staff note how children’s realities at home might drive certain behaviours, particularly when they become stressed or dysregulated. Rather than seeing the behaviour as deliberately defiant, staff model reflection and understanding, aspects which are central to effective trauma informed practices (McCluskey, 2018; Bates, 2021). The notion of parentification (Boszormenyi-Nagy & Spark, 1973; Jurkovic, 1998) became apparent when analysing the staff comments, where their awareness that children might act out as a mechanism to check on the safety of their parents was discussed. This awareness demonstrates the importance of understanding the motivation around a behaviour display, especially for children who have experienced trauma and adversity.

Finally, staff challenged deficit thinking about socioeconomic status and parenting, where whilst acknowledging that deprivation can create barriers, positive, supportive and nurturing parenting is not exclusive to any one socioeconomic group. Teachers’ biases and expectations can influence children’s experiences and in turn outcomes (Gershenson, 2016), therefore an awareness of such biases through continuous professional development that prioritises empathy, cultural awareness, and relationality are key to challenging such assumptions. Again, staff present a strengths-based narrative that emphasises love, routine and parent/carer engagement as protective factors for children. This disrupts and challenges the deterministic link between socioeconomic deprivation and poor educational outcomes (Gershenson et al., 2016).

In conclusion, the findings from the staff interviews reinforce how trauma informed practices promote relational, cultural responsive practices that are grounded in an ethos of support for children and their families. By aligning these practices to the needs of the school community, the school offers a compelling example of how schools can promote meaningful partnerships between home and school.

## Conclusion

The aims of this study were to analyse how school staff perceive partnerships with parents/carers in a trauma informed school. Drawing from qualitative data in a school that identifies as being trauma informed, the findings highlight the ways in which relational, inclusive partnerships are maintained by staff. Rather than viewing parent/carer involvement as peripheral to school culture, the interviewed staff describe these partnerships as central to trauma informed practice, particularly for children who have experienced trauma or adversity in their lives. The findings portray that staff work to construct home school partnerships through practices of empathy, cultural competency and an awareness of deficit-based assumptions. However, these practices are at odds with typical school practices, such as punitive behaviour management strategies which are reinforced through structural inequalities and educational policy.

The study adds to existing literature that frames trauma informed practices as a relational pedagogy for children in schools (Sherwood, 2017; McCluskey, 2018; Bates, 2021) whilst also providing a more nuanced insight into the importance of extending this practice to parents/carers. However, this must not be assumed as a shift that staff and schools can merely adopt as a goodwill gesture, instead a consistent approach, grounded in whole school support and guidance is necessary to ensure children and their families feel valued and accepted in schools (Martin & Collie 2019; Lopes et al., 2017). In current educational contexts, with policy pressure and performativity demands, the difficulty of achieving such consistency must not be underestimated (Thomas, 2021).

## Limitations and Recommendations

Despite the strengths highlighted, the study’s limitations must be acknowledged to support future research. The study was a small scale, context specific inquiry based in a primary school that identified as trauma informed, in an area of high deprivation. The relational culture observed by school staff towards parent/carer partnerships may have been influenced by the school’s context, such as dedicated staff, related training, and supportive leadership, all of which may limit the transferability of findings. The study also focuses exclusively on the voice of school staff, meaning that the bidirectional nature of engagement from the perspective of parents/carers cannot be determined.

For schools seeking to move towards a more trauma informed approach, this study highlights the need for change that is meaningful to the setting and endorsed by leaders. While training provides a useful starting point, the findings suggest that authentic change, that affirms the diverse strengths of families, is more likely when leaders advocate for the immersion of trauma informed practices within school culture. Creating protected time for staff to reflect and receive support on their interactions with children and their families, and to share examples of effective relational approaches, may also support a more consistent understanding across a school community. The study indicates how small, deliberate actions, particularly those supported by leaders, can begin to embed trauma informed values within the wider culture of a school.

## Supplementary Information

Below is the link to the electronic supplementary material.

Supplementary Material 1 (DOCX 54.8 KB)

## Data Availability

No datasets were generated or analysed during the current study.
