# Supplementary material for: Analysing Staff Perspectives on Parent/Carer Partnerships in an English Trauma Informed School
Source: J Child Adolesc Trauma. 2026 Apr 1;19(3):1709–19. doi: 10.1007/s40653-026-00849-8 (PMC13546194; doi:10.1007/s40653-026-00849-8)
Supplement: Supplementary file 1 [file 40653_2026_849_MOESM1_ESM.docx]

Staff Name	Role
Sadie	Emotional Literacy Support Officer [ELSA]
Maeve	Early Years Teaching Assistant
Hayley	Key Stage 2 Teacher
Aisha	Key Stage 2 Teacher
Cara	Emotional Literacy Support Officer [ELSA]
Sam	Key Stage 1 Teacher
Layla	Early Years Teacher
Roz	Key Stage 1 Teaching Assistant

Table 1 – Participant roles within the school.
